# A novel untrained SSVEP-EEG feature enhancement method using canonical correlation analysis and underdamped second-order stochastic resonance

**DOI:** 10.3389/fnins.2023.1246940

**Published:** 2023-10-04

**Authors:** Ruiquan Chen, Guanghua Xu, Huanqing Zhang, Xun Zhang, Baoyu Li, Jiahuan Wang, Sicong Zhang

**Affiliations:** ^1^School of Mechanical Engineering, Xi’an Jiaotong University, Xi’an, China; ^2^State Key Laboratory for Manufacturing Systems Engineering, School of Mechanical Engineering, Xi’an Jiaotong University, Xi’an, China

**Keywords:** motion checkerboard patterns, brain-computer interface, canonical correlation analysis, underdamped second-order stochastic resonance, information transmission rate

## Abstract

**Objective:**

Compared with the light-flashing paradigm, the ring-shaped motion checkerboard patterns avoid uncomfortable flicker or brightness modulation, improving the practical interactivity of brain-computer interface (BCI) applications. However, due to fewer harmonic responses and more concentrated frequency energy elicited by the ring-shaped checkerboard patterns, the mainstream untrained algorithms such as canonical correlation analysis (CCA) and filter bank canonical correlation analysis (FBCCA) methods have poor recognition performance and low information transmission rate (ITR).

**Methods:**

To address this issue, a novel untrained SSVEP-EEG feature enhancement method using CCA and underdamped second-order stochastic resonance (USSR) is proposed to extract electroencephalogram (EEG) features.

**Results:**

In contrast to typical unsupervised dimensionality reduction methods such as common average reference (CAR), principal component analysis (PCA), multidimensional scaling (MDS), and locally linear embedding (LLE), CCA exhibits higher adaptability for SSVEP rhythm components.

**Conclusion:**

This study recruits 42 subjects to evaluate the proposed method and experimental results show that the untrained method can achieve higher detection accuracy and robustness.

**Significance:**

This untrained method provides the possibility of applying a nonlinear model from one-dimensional signals to multi-dimensional signals.

## 1. Introduction

Brain-computer interface (BCI) is a normal output pathway system that does not rely on the composition of peripheral nerves and muscles, and can directly convert central nervous activities into artificial output (Vidal, 1973; Li et al., 2017; Jin et al., 2021).

Steady-state visual evoked potentials (SSVEPs) based on BCI systems have the advantages of short training time, high signal-to-noise ratio, and short response time, and are widely used in clinical detection technology (Li et al., 2022). When an external visual stimulus of constant frequencies is applied, the neural network consistent with the stimulation frequency or harmonic components will generate resonance, causing the brain’s potential activity to change significantly at the stimulation frequency or harmonic components, resulting in SSVEP signals. SSVEP signals can exhibit spectral peaks at stimulation frequency or harmonic components in the power spectrum of EEG signals (Kramer et al., 2021). By analyzing and detecting the frequency corresponding to the spectral peak, it is possible to detect the stimulus source of the subject’s visual gaze, thereby identifying the subject’s intention. However, although SSVEP induced by the motion checkerboard paradigm can reduce visual fatigue in subjects, due to its generation mechanism, there are almost no harmonic components and frequency energy is more concentrative, thus leading to low recognition accuracy (Han et al., 2018).

The first application in SSVEP feature classification is the Power Spectral Density Analysis (PSDA) algorithm (Ming Cheng et al., 2002), which uses the Fast Fourier Transform (FFT) to convert SSVEP signals from the time domain to the frequency domain, thereby obtaining the amplitude and phase characteristics of each stimulus frequency. Because this method only analyzes one electrode signal in multi-channel signals, the obtained signal has a low signal-to-noise ratio (SNR). Wavelet Transform (WT) can be regarded as a Fourier Transform with an adjustable window, which provides both relevant frequency components and occurrence time information, but it still cannot identify nonlinear signals well (Hu et al., 2014). Volosyak et al. proposed the Minimum Energy Combination (MEC) algorithm (Friman et al., 2007), which mainly seeks a spatial filter to project multi-channel signals into low dimensional space. MEC can effectively reduce background noise, but useful information in EEG signals may be lost in the linear transformation. The canonical correlation analysis (CCA) algorithm was first applied to SSVEP classification by Lin et al. (2006). CCA mainly projects multi-channel SSVEP signals and corresponding reference signals into a low dimensional space through a spatial filter and then calculates the correlation between the two. The maximum value of the correlation coefficient corresponds to the stimulation frequency, which is superior to the MEC algorithm. Currently, many variants of the CCA method have achieved excellent BCI performance, such as multi-way canonical correlation analysis (MwayCCA; Zhang et al., 2011), multi-set canonical correlation analysis (MestCCA; Zhang et al., 2014a), Filter bank canonical correlation analysis (FBCCA; Chen and Gao, 2015), Task-related component analysis (TRCA; Nakanishi et al., 2017), Task-discriminant component analysis (TDCA; Liu et al., 2021) and so on. Among them, the FBCCA method is the most effective and widely used untrained method in SSVEP-EEG detection technology. Nevertheless, due to the low harmonic components of SSVEP induced by motion checkerboard patterns, the FBCCA method cannot play its role. In addition, some novel methods, such as multivariate synchronization index (Zhang et al., 2014b), likelihood ratio test (Zhang et al., 2014c), and stochastic resonance analysis (Chen et al., 2021a), have also proven to have unique advantages in SSVEP recognition.

Benzi et al. (1981) first proposed the concept of stochastic resonance (SR) when studying the problem of global glacial periods, and successfully applied it to explain the phenomenon of periodic changes in paleoclimate. Shortly after, Fauve and Heslot (1983) observed the phenomenon of SR while studying the synchronization of noise-induced transitions in a bistable system experiment with a trigger circuit. Mcnamara et al. (1988) once again verified the existence of bistable stochastic resonance (BSR) in the ring laser experiment. Collins et al. (1995) first extended SR theory to the field of aperiodic signal processing when studying FitzHugh Nagumo (FHN) neuron models. Recently, Chen et al. (2021a,b) and Chen et al. (2022) demonstrated in experiments that the FHN neuron model can effectively enhance the feature responses of EEG signals, thereby improving recognition accuracy, regardless of the time domain, frequency domain, or time-frequency domain. Lu et al. (2015) proposed underdamped second-order stochastic resonance (USSR) to improve weak signal detection technology. This novel model considers the system inertia and underdamped damping factor based on bistable stochastic resonance (BSR), which is more conducive to high SNR output. Traditional denoising methods improve the SNR by suppressing noise, which may result in the loss of useful features. However, SR utilizes the synergistic effects of input signals, noise, and resonance systems to enhance feature responses of signals, and has excellent nonlinear signal detection capabilities with noise immunity.

The main contribution of this study is to propose a novel untrained SSVEP feature enhancement method using CCA dimensionality reduction technology and the USSR model. Compared with mainstream unsupervised dimensionality reduction methods, such as common average reference (CAR; Orekhova et al., 2002), principal component analysis (PCA; Wold et al., 1987), multidimensional scaling (MDS; Saeed et al., 2018), and locally linear embedding (LLE; De Ridder et al., 2003), the CCA method reflects a high degree of matching with SSVEP signals. Experimental results show that the CCA-USSR method has higher recognition accuracy, ITR, and better robustness in all subjects.

The rest of this article is arranged as follows: section 2 introduces in detail typical dimensionality reduction methods and the novel CCA-USSR framework proposed in this study. In section 3, the specific experiments and results obtained by different methods are explained. Compared with the CAR, PCA, MDS, and LLE methods, the CCA-USSR method showed a better BCI performance in all subjects. The processing results of each method are discussed in section 4. Finally, section 5 provides the conclusions.

## 2. Methodology

### 2.1. The USSR model and standard FBCCA method

#### 2.1.1. USSR

The Langevin equation for the BSR model is an overdamped first-order differential equation due to neglecting the inertia term and normalizing the damping factor. Nevertheless, it has been proven (Lu et al., 2015) that the system inertia and damping factor can facilitate high-SNR output. Considering these two factors, the BSR model is improved into a second-order differential equation which is called the USSR model. Hence, the USSR model can be expressed as

(1) $$\frac{d^{2}x}{dt^{2}}=-\frac{dU\left(x\right)}{dx}-\beta \frac{dx}{dt}=\mathrm{ax}-bx^{3}-\beta \frac{dx}{dt}+s\left(t\right)+n\left(t\right)$$

where a and b are the system parameters satisfying $a,b\in R^{+}$ , x is the output signal, $s\left(t\right)$ is the input signal. $n\left(t\right)$ is the Gaussian white noise with a mean value of zero and an autocorrelation function satisfying $n\left(t_{1}\right)n\left(t_{2}\right)=2D\delta \left(t_{1}-t_{2}\right)$ (Wu and Zhu, 2008); . represents the overall mean value; 0<β<1 is the damping factor. The fourth-order Runge-Kuta algorithm with fixed steps is used to solve the differential equations with higher solution accuracy. To meet the needs of differential equation calculation, the input signal needs to be transformed into a one-dimensional vector.

#### 2.1.2. FBCCA

As a standard untrained SSVEP recognition algorithm, the FBCCA method uses CCA to calculate the canonical correlation coefficient $\rho_{i}$ of each sub-band signal $\left(X_{i},i=1,2,\ldots ,N\right)$ which is divided via multiple filter banks. The feature discrimination coefficient at the i -th target frequency is obtained by

(2) $$\tilde{\rho }=\overset{n}{\underset{i=1}{\sum }}w\left(i\right)\cdot {\left(\rho_{i}\right)}^{2}$$

where $w\left(i\right)$ is the weight of the i -th sub-band signal which can be obtained by

(3) $$w\left(i\right)=i^{-a}+b,i\in \left[1\;N\right]$$

As previously reported (Chen et al., 2015), a and b constants are set to 1.25 and 0.25.

### 2.2. Typical unsupervised dimensionality reduction methods

Data dimensionality reduction can be used as a means of feature extraction: to identify the main features from the original features of the dataset, that is, the features that best describe the distribution of data in the dataset. In other words, while preserving the main features of the dataset, high-dimensional data is projected into a low-dimensional feature space. Since the input requirement of the USSR model is a one-dimensional vector, a data dimensionality reduction method that matches the EEG rhythm features is required. The following describes five typical unsupervised dimensionality reduction methods.

#### 2.2.1. CAR

The principle is to calculate the average signal of all recording electrodes, and then subtract this average value from the selected reference electrode. However, the method is influenced strongly by high-amplitude artifacts at the selected reference electrode. Therefore, the selection of reference electrodes is crucial for the CAR method. The single-electrode output potential $V_{\mathrm{CAR}}$ between the electrode i and the reference electrode can be expressed as


(4) $$V_{\mathrm{CAR}}=V_{\mathrm{reference}}-\frac{1}{n}\overset{n}{\underset{i=1}{\sum }}V_{i}$$

Where $V_{\mathrm{reference}}$ is the selected reference signal, n is the number of electrodes.

#### 2.2.2. PCA

It is one of the most popular unsupervised linear dimensionality reduction methods nowadays. Its main idea is to obtain a new matrix with the largest variance in the projected dimension after data is multiplied by a matrix, thereby using fewer data dimensions while retaining the characteristics of more original data. The measure of information quantity is the variance of data, which is described as follows


(5) $$Var\left(y_{i}\right)=a_{i}^{T}\overset{m}{\underset{i=1}{\sum }}a_{i}$$

where $a_{i}$ is the i -th transformation vector, Σ is the covariance matrix of the original data, $y_{i}$ is the i -th principal component, $Var\left(y_{i}\right)$ is the variance of the i -th principal component.

The PCA method uses orthogonal transformations to convert the observed data into principal components represented by linear independent variables, the number of which is usually smaller than the number of original variables. Thus, PCA is a common dimensionality reduction method using the linear projection rule.

#### 2.2.3. MDS

It uses the paired similarity of samples to construct a low-dimensional space so that the distance of each pair of samples in the high-dimensional space is as consistent as possible with the sample similarity in the constructed low-dimensional space. A greater similarity between two objects can be reflected by a smaller distance in MDS space. The basic principle is described by


(6) $$\delta_{jk}=\sqrt{\overset{p}{\underset{i=1}{\sum }}{\left(y_{ij}-y_{ik}\right)}^{2}}$$

where $\delta_{jk}$ is the dissimilarity between samples *j* and *k*, *p* is the number of properties used to perform MDS, and ***y*** is the elemental concentration or index in this study.

Hence, classic MDS performs dimensionality reduction on high-dimensional data while ensuring a consistent distance between the original space and low-dimensional spatial samples.

#### 2.2.4. LLE

It is one of the commonly used manifold learning methods and a nonlinear dimensionality reduction method suitable for processing nonlinear data. It is based on the manifold assumption that data in high-dimensional space is distributed on low-dimensional manifolds. The LLE dimensionality reduction method can be described as the following three steps:

1. For each data point $x_{i}$ , find its K nearest neighbors.
2. Compute the reconstruction weights of the neighbors that minimize the error of reconstructing $x_{i}$ .

(7) $$\varepsilon \left(W\right)=\overset{N}{\underset{i=1}{\sum }}x_{i}-\overset{N}{\underset{j=1}{\sum }}w_{ij}{x_{j}}^{2}$$

Subject to $w_{ij}=0$ , if $x_{j}\notin \left\{x_{i}^{1},x_{i}^{2},\ldots ,x_{i}^{K}\right\}$ , and $\overset{N}{\underset{j=1}{\sum }}w_{ij}=1$ .

1. Compute the low-dimensional embedding ***Y*** for $y_{i}$ that best preserves the local geometry represented by the reconstruction weights.

(8) $$\delta \left(Y\right)=\overset{N}{\underset{i=1}{\sum }}y_{i}-\overset{N}{\underset{j=1}{\sum }}w_{ij}{y_{j}}^{2}$$

Subject to $1/N\overset{N}{\underset{i=1}{\sum }}y_{i}y_{i}^{T}=I$ , and $\overset{N}{\underset{i=1}{\sum }}y_{i}=0$ , where 0 is a column vector of zeros and I is an identity matrix. By the Rayleigh-Ritz theorem (Luce and Perry, 1949), minimizing (10) with respect to the $y_{i}$ ’s can be done via finding the eigenvectors with the smallest (nonzero) eigenvalues.

#### 2.2.5. CCA

The projection principle selected by the CCA method is that after dimensionality reduction, the correlation coefficient of the two sets of data is the largest. For input EEG data X and the reference signal Y , the goal of CCA is to find weight vectors $w_{x}$ and $w_{y}$ , so that the one-dimensional vectors obtained after X and Y projection are $X^{\prime }$ and $Y^{\prime }$ , respectively. Therefore, one-dimensional vectors are obtained by


$$X^{\prime }={w_{x}}^{T}X$$

(9) $$Y^{\prime }={w_{y}}^{T}Y$$

The optimization goal of the CCA dimensionality reduction method is to maximize $\rho \left(X^{\prime },Y^{\prime }\right)$ to obtain the corresponding projection vectors $w_{x}$ and $w_{y}$ . Then the correlation coefficient ρ between weight vectors $w_{x}$ and $w_{y}$ was calculated by


(10) $$\max_{w_{x}w_{y}},\rho \left(X^{\prime },Y^{\prime }\right)=\frac{E\left[w_{x}^{T}XY{\left(f\right)}^{T}w_{y}\right]}{\sqrt{E\left[w_{x}^{T}XX^{T}w_{x}\right]}E\left[\begin{array}{l} w_{y}^{T}Y\left(f\right)\\ Y{\left(f\right)}^{T}w_{y} \end{array}\right]}$$

The frequency corresponding to the maximum correlation coefficient is regarded as the gaze target of the subjects.

The template signals of the CCA method are given by


(11) $$Y\left(f_{d}\right)=\left\{\begin{array}{c} \cos\left(2\pi f_{d}t\right)\\ \sin\left(2\pi f_{d}t\right)\\ \ldots \\ \cos\left(2k\pi f_{d}t\right)\\ \sin\left(2k\pi f_{d}t\right) \end{array}\right\},t=1/f_{s},\ldots ,N/f_{s}$$

where *k* is the number of harmonics of SSVEP signals; $f_{d}$ is the stimulation frequency of the template signals; *N* is the number of sample points; $f_{s}$ is the sampling frequency.

### 2.3. Feature enhancement for SSVEP using canonical correlation analysis and underdamped second-order stochastic resonance

**Figure 1:**
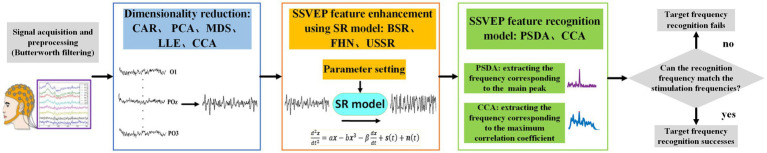
The flowchart of SSVEP detection using the CCA dimensionality reduction method and USSR model.

When the human eye receives a fixed frequency of visual stimulation, the potential activity of the cerebral cortex will be modulated to produce a continuous response related to the stimulation frequency. This response has a periodic rhythm similar to the visual stimulation, that is, the steady-state visual evoked potential. The SSVEP signal can exhibit spectral peaks at the stimulus frequency or harmonic components in the power spectrum. By analyzing the frequency corresponding to the spectral peak, the stimulus source of the subject’s visual gaze can be detected, thereby identifying the subject’s intention. The novel untrained framework based on the CCA dimensionality reduction method and USSR model proposed in this study is shown in Figure 1. Detailed procedures are described as follows:

- Signal acquisition and preprocessing. Since the raw SSVEP signals are usually weak and mixed with multi-scale noise, it is difficult to extract gaze frequencies in a single trial. Some preprocessing steps such as filtering techniques need to be used to remove noise interference. As previously described (Han et al., 2018), since the motion checkerboard pattern has few harmonic components, in our study, we only consider the fundamental frequency and the primary harmonic to achieve the highest ITR. Hence, a Butterworth filter with a passband range of 3–40 Hz is selected to remove noise and some high-frequency components.
- Dimensionality reduction. Although the SR model can effectively enhance the feature frequency of SSVEP signals, due to the characteristic of its differential equation, the input signal needs to be transformed into a one-dimensional vector. Common unsupervised dimensionality reduction methods include CAR, PCA, MDS, LLE, and CCA. In our study, these methods are compared to get the optimal dimensionality reduction methods. In the CAR method, we choose the Oz channel as the reference channel. The experimental results indicate that the Oz channel has the highest recognition accuracy compared with other channels. In the LLE method, the number of nearest neighbor points is set to 40.
- SSVEP feature enhancement. Typical SR models include BSR, FHN, and USSR, among which the USSR model has the best BCI performance despite having the most parameters (Chen et al., 2023). Therefore, in this study, the USSR model was used as a means of feature enhancement.
- SSVEP feature recognition. Then, PSDA and CCA recognition methods are used to identify the subjects’ gaze targets.
- Target discrimination. Finally, the recognition accuracy is obtained by matching the recognition frequency with the stimulus frequency.

## 3. Experiment and results

### 3.1. Experiments and datasets

The experiment data included 30 males and 12 females (42 subjects, average age ± SD, 27.2 ± 2.6) originating from Chen et al. (2023). Each subject has a normal or corrected vision.

**Figure 2:**
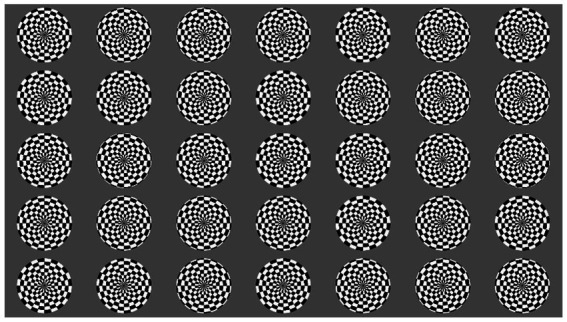
The user interface of the 35 focused targets.

**Figure 3:**
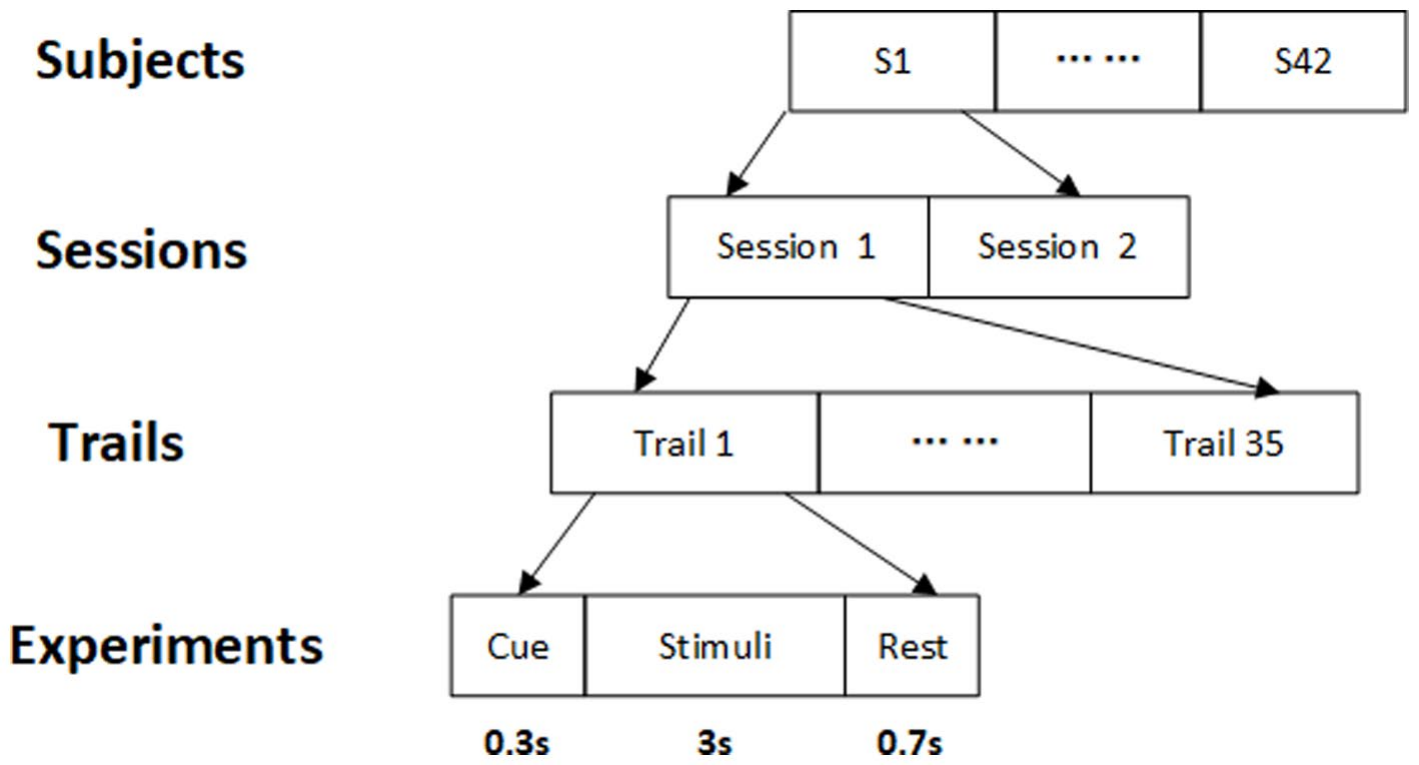
The experimental process.

The ring-shaped checkerboards with radial contraction–expansion motion were adopted as the visual stimuli in our experiment. The stimulus paradigm was arranged into a 5 × 7 matrix with a horizontal and vertical separation of 100 pixels and 50 pixels between two adjacent stimuli, respectively. The frequency range of 35 focused targets was 3–20 Hz with a frequency interval of 0.5 Hz. The 35 focused frequencies for each trial were presented simultaneously with the data sampled at 1,000 Hz, as shown in Figure 2. The g.USBamp (g.tec Inc., Austria) was utilized to record SSVEP signals and the channels were set according to the 10/20 electrode system. These eight electrodes POz, PO3, PO4, PO5, PO6, Oz, O1, and O2 were used to record the raw SSVEP signal. SSVEP is a specific EEG signal generated by the occipital region of the brain. These eight electrodes are located closest to the occipital lobe, so the signals collected by them are less noisy and more stable. Each subject is required to conduct 35 trials, each consisting of 0.3 s of cues, 3 s of visual stimuli, and 0.7 s of rest time. The experimental conditions can be found in the study (Chen et al., 2021a). The experimental process is displayed in Figure 3.

### 3.2. CCA coefficient spectrums analysis for SSVEP

**Figure 4:**
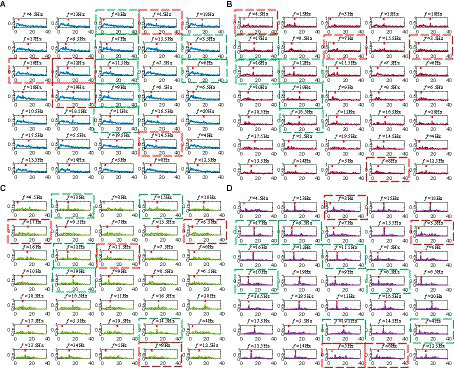
**(A)** The coefficient spectrums of the CCA method. **(B)** The coefficient spectrums of the PCA-USSR method. **(C)** The coefficient spectrums of the LLE-USSR method. **(D)** The coefficient spectrums of the CCA-USSR method.

Based on the above analysis, we know that the SR model can utilize the noise energy to enhance the target frequency through synergistic effects and noise immunity. Therefore, the main research direction of this paper is to preserve as many effective features as possible in the original multi-channel signals. As representative dimensionality reduction methods among the eight methods, we compared the coefficient spectrums of CCA, PCA-USSR, LLE-USSR, and CCA-USSR in Figure 4. In this study, the representative subject (S29) with the 2 s data length was utilized to compare the BCI performance. The correlation coefficient between the processed SSVEP signal and the reference signal at a 0.5 frequency interval of 1-40 Hz is described as the CCA coefficient spectrum in our study.

The CCA coefficient spectrum between the filtered EEG and the template is presented in Figure 4A. As a classic SSVEP recognition method, the CCA method can effectively identify the target frequency of most subjects. For example, the CCA coefficients corresponding to the seven target frequencies of 3, 7, 3.5, 11.5, 6, 9, and 11 Hz (the CCA spectrums marked by the green box) have the maximum amplitude in the entire spectrum and the recognition succeeds. However, it is worth noting that in these successful cases, the amplitudes of several interference peaks are very close to that of the target frequencies, making the recognition effect not ideal. On the other hand, the CCA coefficients corresponding to the five frequencies of 15, 16, 12, 19, and 14.5 Hz (the CCA spectrums marked by red boxes) have not the maximum amplitude in the CCA spectrum, and their amplitudes are second only to the maximum interference peak. Hence, the feature extraction for SSVEP recognition finally fails and there is a need for new methods to enhance the energy of the target frequency and improve the BCI decoding performance under the motion checkerboard pattern.

According to the previous study (Yao et al., 2019), the optimal parameter combination for the USSR model is [a, b, 𝛽, h] = [0.1, 1, 0.35, 0.1].

Compared with the CCA method, the USSR-based methods reduce the amplitude of interference peaks in the CCA coefficient spectrum and increase the energy of the target frequency, making BCI recognition more accurate. The amplitude of target frequencies depends on the matching between the dimensionality reduction method and the SSVEP rhythm components. As shown in Figure 4B, in the corresponding coefficient spectrums at 17, 16, 12, 19, and 18.5 Hz (the coefficient spectrums marked in green boxes), which cannot be correctly identified by the CCA method, the amplitudes of the focused frequencies are enhanced by the PCA-USSR model and have exceeded the amplitude of the interference peaks. However, for the corresponding coefficient spectrums of 4.5, 7, 3.5, and 8 Hz (the coefficient spectrums marked in red boxes), the PCA-USSR method cannot accurately identify target frequencies. As shown in Figure 4C, the combination of the nonlinear LLE method and the USSR model has similar results compared with the linear dimensionality reduction method. For target frequencies that cannot be identified by the CCA method, some can be corrected by the LLE-USSR method, while others cannot be identified.

As shown in Figure 4D, compared with the typical PCA-USSR and LLE-USSR methods, the CCA-USSR method can significantly increase the amplitude of the target frequency and has an energy concentration effect that matches the checkerboard pattern. Therefore, compared with the CCA method, the recognition accuracy of target frequency has increased by approximately 43%. For example, for the seven frequencies of 17, 9.5, 11.5, 10, 8.5, 19.5, and 4 Hz that cannot be identified by the LLE-USSR method, the USSR model can further enhance the energy of the target frequency and optimize the SSVEP recognition performance. While, in the power spectrums corresponding to 3, 3.5, 5, and 8 Hz, since the CCA dimensionality reduction method does not retain the effective features of the original SSVEP well, the USSR model incorrectly enhances the energy of other frequencies, resulting in recognition failure.

The above analysis has demonstrated that the CCA dimensionality reduction method can preserve the effective features of the original signal to the greatest extent and the nonlinear weak feature enhancement based on USSR dynamics models is highly compatible with the non-stationary SSVEP. The USSR model takes advantage of the unique conversion of noise energy to signal energy, thereby enhancing the amplitude and the energy of focused targets and improving the BCI decoding performance. Table 1 shows the average accuracy and ITR of 42 subjects based on five dimensionality reduction methods and two standard methods for processing SSVEP signals (Data length T = 2 s).

**Table 1:** Detection performance using the five methods (T = 2 s).

Subjects	Accuracy % (CCA)	ITR bits/min (CCA)	Accuracy % (FBCCA)	ITR bits/min (FBCCA)	Accuracy % (CAR-USSR)	ITR bits/min (CAR-USSR)	Accuracy % (PCA-USSR)	ITR bits/min (PCA-USSR)
S1	94.29	135.68	82.86	107.89	8.57	1.68	54.29	54.27
S2	28.57	18.97	34.29	25.76	80.00	101.70	42.86	37.11
S3	77.14	95.73	68.57	78.97	74.29	89.96	74.29	89.96
S4	48.57	45.40	54.29	54.27	57.14	58.91	48.57	45.40
S5	88.57	121.05	65.71	73.72	54.29	54.27	22.86	12.87
S6	94.29	135.68	77.14	95.73	88.57	121.05	77.14	95.73
S7	74.29	89.96	82.86	107.89	20.00	10.12	57.14	58.91
S8	77.14	95.73	65.71	73.72	5.71	0.50	62.86	68.64
S9	68.57	78.97	60.00	63.70	88.57	121.05	54.29	54.27
S10	34.29	25.76	40.00	33.18	77.14	95.73	37.14	29.39
S11	42.86	37.11	57.14	58.91	62.86	68.64	45.71	41.18
S12	34.29	25.76	68.57	78.97	25.71	15.83	71.43	84.38
S13	31.43	22.28	45.71	41.18	28.57	18.97	65.71	73.72
S14	57.14	58.91	65.71	73.72	77.14	95.73	60.00	63.70
S15	42.86	37.11	71.43	84.38	28.57	18.97	71.43	84.38
S16	62.86	68.64	62.86	68.64	82.86	107.89	85.71	114.32
S17	74.29	89.96	62.86	68.64	82.86	107.89	77.14	95.73
S18	62.86	68.64	65.71	73.72	74.29	89.96	57.14	58.91
S19	85.71	114.32	74.29	89.96	94.29	135.68	65.71	73.72
S20	74.29	89.96	65.71	73.72	2.86	0	37.14	29.39
S21	60.00	63.70	65.71	73.72	51.43	49.76	77.14	95.73
S22	68.57	78.97	68.57	78.97	94.29	135.68	65.71	73.72
S23	65.71	73.72	71.43	84.38	88.57	121.05	48.57	45.40
S24	82.86	107.89	91.43	128.14	85.71	114.32	74.29	89.96
S25	51.43	49.76	60.00	63.70	45.71	41.18	37.14	29.39
S26	74.29	89.96	74.29	89.96	31.43	22.28	65.71	73.72
S27	62.86	68.64	60.00	63.70	8.57	1.68	60.00	63.70
S28	68.57	78.97	71.43	84.38	25.71	15.83	42.86	37.11
S29	40.00	33.18	80.00	101.70	82.86	107.89	57.14	58.91
S30	11.43	3.32	40.00	33.18	8.57	1.68	45.71	41.18
S31	62.86	68.64	68.57	78.97	2.86	0	37.14	29.39
S32	91.43	128.14	77.14	95.73	94.29	135.68	82.86	107.89
S33	40.00	33.18	77.14	95.73	85.71	114.32	28.57	18.97
S34	85.71	114.32	77.14	95.73	17.14	7.59	20.00	10.12
S35	71.43	84.38	77.14	95.73	57.14	58.91	60.00	63.70
S36	68.57	78.97	68.57	78.97	2.86	0	62.86	68.64
S37	54.29	54.27	65.71	73.72	82.86	107.89	54.29	54.27
S38	51.43	49.76	60.00	63.70	71.43	84.38	68.57	78.97
S39	40.00	33.18	62.86	68.64	8.57	1.68	31.43	22.28
S40	42.86	37.11	54.29	54.27	77.14	95.73	17.14	7.59
S41	57.14	58.91	65.71	73.72	5.71	0.50	40.00	33.18
S42	62.86	68.64	62.86	68.64	57.14	58.91	62.86	68.64
Mean ± SD	61.16 ± 19.59	66.68 ± 33.48	65.99 ± 11.68	74.22 ± 20.66	52.38 ± 32.75	51.25 ± 48.71	54.97 ± 17.38	55.36 ± 27.32

Subjects	Accuracy % (MDS-USSR)	ITR bits/min (MDS-USSR)	Accuracy % (LLE-USSR)	ITR bits/min (LLE-USSR)	Accuracy % (CCA-USSR)	ITR bits/min (CCA-USSR)
S1	57.14	58.91	65.71	73.72	85.71	114.32
S2	34.29	25.76	25.71	15.83	54.29	54.27
S3	74.29	89.96	54.29	54.27	74.29	89.96
S4	54.29	54.27	62.86	68.64	57.14	58.91
S5	57.14	58.91	40.00	33.18	77.14	95.73
S6	82.86	107.89	65.71	73.72	88.57	121.05
S7	60.00	63.70	42.86	37.11	82.86	107.89
S8	60.00	63.70	51.43	49.76	71.43	84.38
S9	54.29	54.27	62.86	68.64	62.86	68.64
S10	37.14	29.39	42.86	37.11	51.43	49.76
S11	51.43	49.76	28.57	18.97	60.00	63.70
S12	71.43	84.38	60.00	63.70	77.14	95.73
S13	65.71	73.72	45.71	41.18	65.71	73.72
S14	54.29	54.27	40.00	33.18	77.14	95.73
S15	77.14	95.73	45.71	41.18	80.00	101.70
S16	85.71	114.32	77.14	95.73	88.57	121.05
S17	82.86	107.89	51.43	49.76	77.14	95.73
S18	57.14	58.91	37.14	29.39	62.86	68.64
S19	62.86	68.64	42.86	37.11	88.57	121.05
S20	68.57	78.97	42.86	37.11	74.29	89.96
S21	68.57	78.97	68.57	78.97	77.14	95.73
S22	65.71	73.72	62.86	68.64	82.86	107.89
S23	45.71	41.18	51.43	49.76	65.71	73.72
S24	77.14	95.73	65.71	73.72	88.57	121.05
S25	40.00	33.18	51.43	49.76	65.71	73.72
S26	71.43	84.38	60.00	63.70	80.00	101.70
S27	65.71	73.72	45.71	41.18	80.00	101.70
S28	42.86	37.11	42.86	37.11	80.00	101.70
S29	62.86	68.64	68.57	78.97	82.86	107.89
S30	51.43	49.76	28.57	18.97	54.29	54.27
S31	45.71	41.18	25.71	15.83	77.14	95.73
S32	80.00	101.70	65.71	73.72	88.57	121.05
S33	28.57	18.97	37.14	29.39	68.57	78.97
S34	28.57	18.97	17.14	7.59	82.86	107.89
S35	62.86	68.64	28.57	18.97	85.71	114.32
S36	71.43	84.38	57.14	58.91	74.29	89.96
S37	57.14	58.91	54.29	54.27	80.00	101.70
S38	74.29	89.96	62.86	68.64	71.43	84.38
S39	25.71	15.83	28.57	18.97	65.71	73.72
S40	22.86	12.87	57.14	58.91	68.57	78.97
S41	54.29	54.27	25.71	15.83	60.00	63.70
S42	68.57	78.97	42.86	37.11	71.43	84.38
Mean ± SD	58.57 ± 16.29	61.29 ± 26.45	48.44 ± 14.73	45.20 ± 21.77	74.01 ± 10.42	89.42 ± 20.35

### 3.3. BCI performance

Here, paired *t*-tests were performed to determine significant differences (defined as *p* < 0.05) in accuracy and ITR for different methods. The information transfer rate (ITR) is an important and effective indicator to measure SSVEP-BCI recognition performance among different methods. It is used to express the amount of information transmitted in a unit of time. ITR can be obtained by


(12) $$ITR=\frac{60}{T}\left[\log_{2}M+\sigma \log_{2}\sigma +\left(1-\sigma \right)\log_{2}\left(\frac{1-\sigma }{M-1}\right)\right]$$

where *σ* refers to the average recognition accuracy, *M* refers to the number of gaze frequencies and *T* refers to the data length for analysis.

The higher the average recognition accuracy, the larger the number of gaze targets, the shorter the used data length, and the higher the obtained ITR. Meanwhile, the data length also affects recognition accuracy. For example, too short a data length may result in fewer recognizable SSVEP features and a decrease in recognition accuracy.

Using the classic CCA method to identify the gaze frequencies of 42 subjects can achieve an average accuracy of 61.16 ± 19.59 and an ITR of 66.68 ± 33.48. As the state-of-the-art method for SSVEP recognition, the average accuracy and ITR of FBCCA are increased to 65.99 ± 11.68 and 74.22 ± 20.66 bits/min, respectively.

It is worth noting that the CAR-USSR method is not only affected by the reference channel but also has the worst robustness among the five dimensionality reduction methods with a variance of 48.71 in the ITR. Nevertheless, there is no significant difference between the CAR-USSR, PCA-USSR, MDS-USSR, and LLE-USSR methods (*p* > 0.05).

As a representative of nonlinear manifold learning methods, the recognition accuracy of the LLE-USSR method greatly depends on the number of nearest neighbors. Meanwhile, from the experimental results, it can be seen that although the number of nearest neighbors is set to 40, the LLE-USSR method has not achieved competitive BCI performance. Compared with other dimensionality reduction methods, CCA can retain the most features of multi-channel SSVEP signals and the CCA-USSR method has the highest recognition accuracy, ITR, and robustness (*p* < 0.05). Meanwhile, compared with typical CCA and FBCCA methods, the CCA-USSR method is also more suitable for SSVEP signals induced by the motion checkerboard paradigm (*p* < 0.05). Hence, we can conclude that CCA is currently the best dimensionality reduction method in EEG signals, and the untrained CCA-USSR method can achieve satisfactory results in real-time BCI applications and spectral analysis.

**Figure 5:**
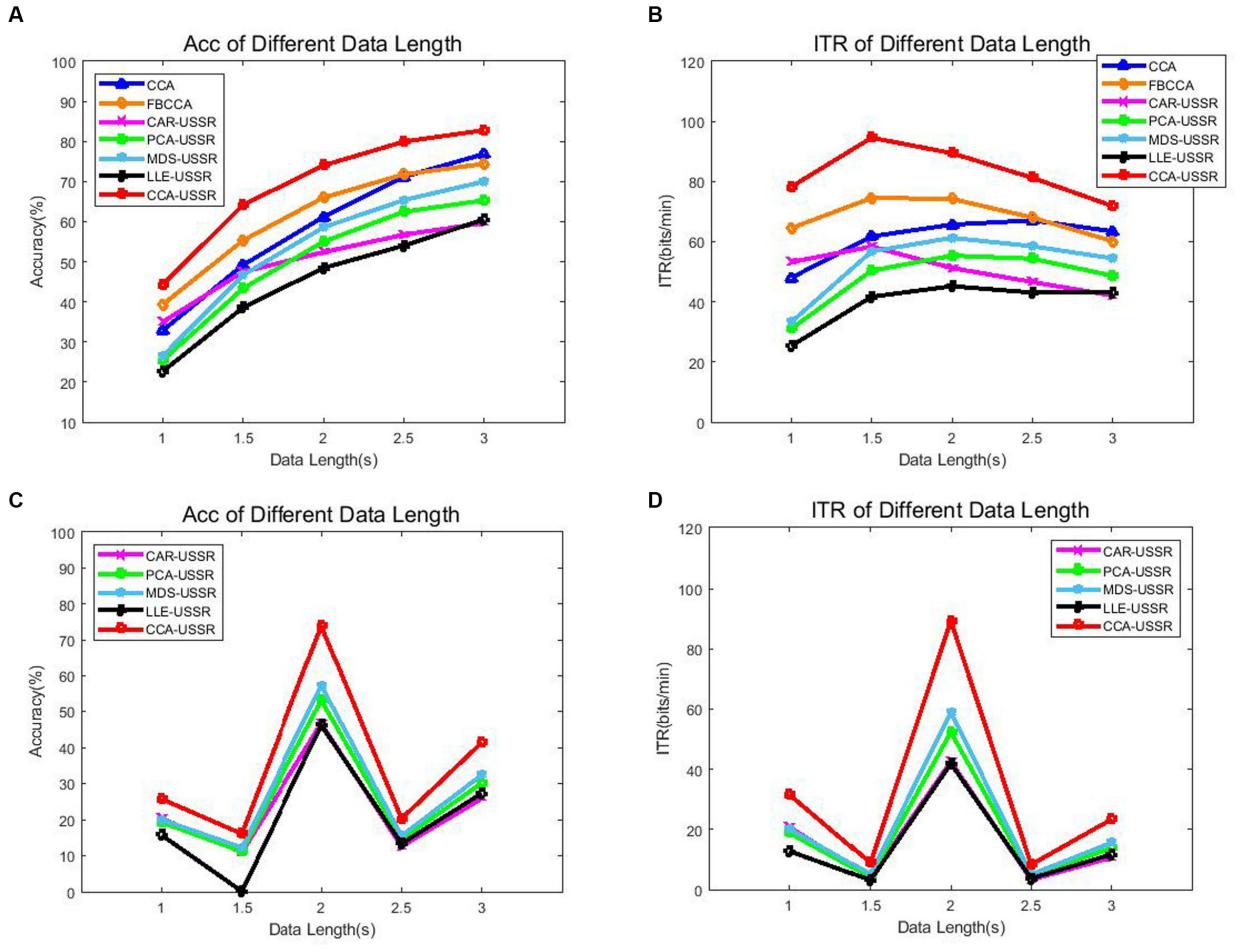
**(A)** Average accuracy using the CCA recognition method. **(B)** Average ITR using the CCA recognition method. **(C)** Average accuracy using the PSDA recognition method. **(D)** Average ITR using the PSDA recognition method.

The analysis data length of SSVEP signals is also an important indicator that significantly affects recognition accuracy and ITR. Here, Figure 5 compares the average recognition accuracy and ITR of CCA, FBCCA, and USSR-based methods under different data lengths (3, 2.5, 2, 1.5, and 1 s).

From Figure 5, each method exhibits reliable results compared with the previous study (Yan et al., 2021), and among these methods, the CCA-USSR method has the highest recognition accuracy and ITR from 1 to 3 s data length. Note that due to the lack of harmonic components in the SSVEP signal induced by the motion checkerboard paradigm, the FBCCA method did not achieve ideal results in our study. Especially, based on PSDA recognition methods, all USSR-based methods can achieve the best BCI decoding performance only at 2 s date length. The experimental results show that the CCA-USSR method can significantly outperform the classic CCA and FBCCA methods, as well as other USSR-based methods, at any data length (*p* < 0.05). This also indicates that the CCA method has a high degree of matching with SSVEP, which can retain the most features of the original multi-channel signal after dimensionality reduction. In addition, the CAR-USSR method has excellent BCI performance comparable to the classic CCA method at 1 s data length. One possible explanation is that CAR relies on electrode selection, so when the data length is shorter, the CAR-USSR method can show better BCI decoding ability. On the other hand, it is not difficult to infer that the nonlinear LLE method is not suitable for non-stationary SSVEP signals. One hypothesis is that multi-channel SSVEP signals do not meet the manifold distribution assumption of input signals. The rationale we get from the experimental results is that although multi-scale noise may have negative effects on SSVEP recognition, the CCA dimensionality reduction method can retain the most features of the original multi-channel SSVEP signal, and the USSR model can use noise energy to enhance the amplitude of the target frequency, thereby improving the detection accuracy and ITR of SSVEP decoding.

Besides, the processing time of unsupervised dimensionality reduction methods was detected to further compare the online calculation ability of different methods in Table 2.

**Table 2:** Comparison of the processing time of five unsupervised dimensionality reduction methods.

	Data length				
	3 s	2.5 s	2 s	1.5 s	1 s
CAR	0.0013 s	0.0012 s	0.0012 s	0.0011 s	0.0010 s
PCA	0.0025 s	0.0023 s	0.0021 s	0.0019 s	0.0017 s
MDS	5.1042 s	2.7754 s	1.4109 s	0.4317 s	0.1669 s
LLE	9.9095 s	6.2133 s	3.6962 s	1.1774 s	0.3849 s
CCA	0.0637 s	0.0615 s	0.05942 s	0.05776 s	0.0543 s

From Table 2, we can see that the CAR and PCA methods have the shortest processing time and the CCA method comes next, while the MDS and LLE methods have the slowest processing speed. In particular, the processing time of the LLE method is determined by the number of nearest neighbor points. The larger the number of nearest neighbor points, the higher the recognition accuracy, but the processing time also increases exponentially. The above results suggest that the CCA-USSR method is suitable for real-time SSVEP detection technology and neuroscience.

## 4. Discussion

This study discusses the impact of different dimensionality reduction methods on multi-channel SSVEP signals. Five typical unsupervised methods were compared from the perspective of the CCA coefficient spectrum, recognition accuracy, ITR, robustness, and processing speed. In the CAR method, the selection of channels is particularly important. The experimental results found that among these eight electrodes POz, PO3, PO4, PO5, PO6, Oz, O1, and O2, using Oz as the reference channel can achieve the highest recognition accuracy and ITR. This also indicates that the Oz channel which is located over the occipital region retains the most effective features of SSVEP signals. In linear dimensionality reduction methods such as CAR, PCA, MDS, and CCA, although they have different rules for projecting high-dimensional data to low-dimensional space, CCA shows the best adaptability to SSVEP signals and MDS comes next. In nonlinear LLE methods, the number of nearest neighbor points determines the quality of detection results and processing speed. The larger the number of nearest neighbor points, the higher the recognition accuracy and ITR, and the longer the processing time. However, due to the strong limitations of the LLE method, which assumes that the input data satisfies the manifold distribution, it is not suitable for extracting the features of SSVEP data. In terms of processing time, CAR, PCA, and CCA are promising for real-time BCI detection technology, while MDS and LLE need more time for processing BCI.

Only when the dimensionality reduction method retains the most features of the original multi-channel signal, the USSR model can more effectively utilize synergistic effects to enhance the energy and amplitude of target frequencies, thereby increasing ITR. The reason why SR is different from other traditional denoising methods is that the SR model utilizes a dynamic feature enhancement mechanism with the help of the synergetic action of input aperiodic signal, noise, and the nonlinear resonance system. The SR model considers noise as a positive factor, thereby using noise energy to enhance weak signal features. In addition, compared with the first-order bistable stochastic resonance, the underdamped second-order SR considers the inertia term and normalizes the damping factor. To use an analogy, first-order BSR processing means primary filtering and second-order USSR processing means secondary filtering, thereby producing a cleaner filtered response than first-order SR. This is the reason why the USSR can more effectively improve the weak signal detection performance than BSR and other classic linear methods.

Although the motion checkerboard paradigm can effectively reduce the fatigue of subjects and is more suitable for long-time BCI performance detection, its evoked SSVEP signal has fewer harmonic components and more concentrated frequency energy (Han et al., 2018). Therefore, some standard untrained algorithms, such as CCA and FBCCA, are not effective in detecting the subject’s purpose (Yan et al., 2021). In addition, using some traditional linear methods to decode SSVEP signals, the useful features will be attenuated or lost while denoising, which seriously affects the improvement of recognition accuracy. Nevertheless, the nonlinear SR model has a frequency energy concentration effect similar to the motion checkerboard pattern. And among typical SR models, the USSR model has the best performance. Naturally, the study proposes to combine dimensionality reduction methods and nonlinear USSR models to extract non-stationary SSVEP features. The experimental results indicate that among traditional dimensionality reduction methods, the CCA method still has the projecting rule that best matches the SSVEP rhythm, which can retain the most original multi-channel SSVEP features, and the USSR model can effectively highlight the energy and amplitude of the target frequency with noise immunity, thereby increasing the algorithm robustness, recognition accuracy, and ITR. This untrained method also provides the possibility of applying a nonlinear model from one-dimensional signals to multi-dimensional signals.

## 5. Conclusion

In this study, we first compare five typical unsupervised dimensionality reduction methods, namely CAR, PCA, MDS, LLE, and CCA. The experimental results show that CCA has the highest adaptability for SSVEP rhythms and can retain the most effective features. Furthermore, compared with the standard CCA and FBCCA methods, the novel untrained CCA-USSR method proposed in this paper can more effectively highlight the target frequency and have higher robustness, thereby increasing recognition accuracy and ITR. In addition, the CCA-USSR method also has advantages in processing speed and has the potential for real-time BCI detection technology.

## Data availability statement

The data analyzed in this study is subject to the following licenses/restrictions: the data is available from the corresponding author upon reasonable request. Requests to access these datasets should be directed to GX, ghxu@mail.xjtu.edu.cn.

## Ethics statement

Ethical approval was not required for the study involving human samples in accordance with the local legislation and institutional requirements because [reason ethics approval was not required]. Written informed consent for participation in this study was provided by the participants’ legal guardians/next of kin.

## Author contributions

RC: conceptualization, methodology, software, formal analysis, data curation, writing—original draft, and writing—review and editing. GX: validation, formal analysis, and investigation. HZ: software. XZ and BL: supervision. JW: funding acquisition. SZ: project administration. All authors contributed to the article and approved the submitted version.
